# Circular bioeconomy: policy and regulatory impacts on livestock production systems

**DOI:** 10.1093/af/vfaf025

**Published:** 2025-09-19

**Authors:** Maja Arsic, Eleazar Gonzalez, Jesus Mendez, Duncan Rowland, Philippe Becquet

**Affiliations:** CSIRO Agriculture and Food, Queensland Biosciences Precinct, St Lucia, QLD, Australia; Lincoln University of Missouri, College of Agriculture, Environment and Human Sciences (CAEHS), Jefferson City, MO, USA; CESFAC, Madrid, Spain; Stock Feed Manufacturers’ Council of Australia (SFMCA), Curtin, ACT, Australia; Philippe Becquet EI, Mulhouse, France

**Keywords:** bioeconomy, circular, livestock, policy, regulatory

**Implications**

- Currently, bioeconomy and circular economy policies are being developed independently in most countries, leading to contradictions and a lack of coherence. It is advisable to combine both into circular bioeconomy policies, allowing for the most efficient and sustainable use of biological resources.
- Implementation of the One Health, EcoHealth, and Planetary Health within these circular bioeconomy policies should be considered to support the sustainable and safe use of resources in livestock production systems and in broader supply chains.
- Regulations and standards aimed at controlling and monitoring the safety of food and waste should encompass the use of byproducts from the livestock production systems, ensuring the safety of animal-sourced products. Implementation of Hazard Analysis and Critical Control Points encompassing the co-products is advisable.

## Introduction

The evolution of the current linear production system into a circular approach, with the use of increased quantities of biomass, requires the development of appropriate policies to encourage and potentially incentivize the circular use of biomass. This development will lead to both the potential use of new materials and allow for the safe use of currently wasted resources from sectors such as livestock production systems, including processing wastes (e.g., whey, wool, wool grease, wastewaters) or manures to produce products, such as feed and fertilizers. It will require a thorough risk assessment to ensure safe use for animals, the environment, and the consumers of edible products. This paper considers the broad governance, policy, and regulation landscape of current national approaches to bioeconomy and circular economy, and the implications for circular bioeconomy approaches for livestock systems. Recommendations are provided to shift towards coherent policies and regulatory systems that address both bioeconomy and circular economy aspects and integrate other principles critical for developing safe and viable circular bioproducts (e.g., Planetary Boundaries and the One Health approach).

## Policies

Circular bioeconomy policies are commonly divided into two parts that need to be considered jointly when evaluating the role of livestock production systems in the circular bioeconomy: bioeconomy policies and circular economy policies.

### Bioeconomy policies

Currently, more than 60 countries have developed bioeconomy-related policies, more than 24 having developed dedicated bioeconomy policies (International Advisory Council on Global Bioeconomy, 2020). While in most countries, bioeconomy policies consider all potential local sources of biomass, some nations have focused on specific resources such as marine (e.g., Portugal) or forestry (e.g., Finland, Canada). Bioeconomy policies may focus on the end-products of biomass processing, typically the production of bioenergy and biofuels, or on the further prospecting of biomass sources, considering traditional bioresources and indigenous knowledge. However, a limited number of countries are integrating their bioeconomy policies into other policies such as circular economy, research and development, and rural development policies (Haarich and Kirchmayr-Novak, 2022).

Depending on the region or country, various key elements are included in bioeconomy policies. Bioeconomy policies often have a heavy focus on sustainable biomass production in forestry and cropping sectors, with less consideration of the role of livestock in a sustainable bioeconomy (Priefer et al., 2017; Muscat et al., 2021). However, livestock can upcycle biomass and produce high-value co-products and should therefore be addressed explicitly in this policy area (Blair et al., 2024). One of the general goals of bioeconomy policies consists of the improvement of knowledge around the potential use of various biomass resources (Priefer et al., 2017). As an example, the “South African Farmer to Pharma” concept promotes the acquisition of indigenous knowledge, the research, and innovation for the development of pharmaceutical specialties (Department of Science and Technology, 2013). The use of local biomass reduces the dependence on non-renewable sources, increases natural resources productivity, and supports regional and rural development (McCormick and Kautto, 2013; International Advisory Council on Global Bioeconomy, 2020; Muscat et al., 2021). However, ensuring that food security and the use of biomass are complementary is necessary, and the use of biomass for food, including animal-sourced food, should be given priority (Muscat et al., 2021).

Some bioeconomy policies and approaches have been criticized if biomass production is considered as inherently sustainable. However, some natural resources could be reoriented (e.g., reduction of feed production due to the diversion of biomass [e.g., dried beet pulp] to produce bioenergy [Madelrieux et al., 2022]) or impact biodiversity (e.g., intensive cultivation of oil palms for biofuel impacts tropical rainforest biodiversity [Issa et al., 2019]). With the objective of further developing national bioeconomy policies, FAO (2021) defined the boundaries of bioeconomy policies that would benefit the communities and the environment, promoting linkages between sectors to support global food and nutrition security.

### Circular economy policies

Circular economy policies aim to use resources efficiently and sustainably by reducing waste production, recycling, and promoting opportunities to utilize all resources at their highest value (Reichel et al., 2016). At least 58 nations currently reference circular economy in policies, policy instruments, or industry sector plans (Haswell et al., 2024). However, in some countries, conflicting regulation on waste management precludes “waste” resources further use for other purposes, and their destruction is mandated (OECD, 2018). Shifting away from waste management toward circular economy helps to improve the efficient use of resources, reduces dependence on natural resources, and thus the extraction and demand for primary natural resources (Bezema, 2016; Reichel et al., 2016; Stegmann et al., 2020).

The development of a circular economy requires some key enabling factors, such as the potential to recycle the product at the end of its life, the introduction of taxes and other policy instruments to discourage pollution, and the removal of incentives on the production of end-of-life products (Reichel et al., 2016). In the food chain, circularity requires collaboration and transparency, supporting the use of material cascades (Bezema, 2016; OECD, 2018), data monitoring, and development of circularity indicators (Reichel et al., 2016).

Regulations specific to certain sectors aim to implement these policies and establish a hierarchy for waste, based on prevention, reuse, recycling, recovery, and disposal, where the use of co-products is first dedicated to the product with the highest societal value (Bezema, 2016; Reichel et al., 2016; Priefer et al., 2017; Muscat et al., 2021), such as healthy supply of food and feed. However, policy instruments (such as regulations and standards) can also act as barriers to developing circular bioeconomy technologies and products due to conflicting requirements for supply chain and product safety and quality (APEC, 2024).

### Circular bioeconomy

Currently, no specific circular bioeconomy-specific policies exist. Thus, it is necessary to refer to both circular- and bioeconomy-specific policies. However, some contradictions exist between these two policy forums. As an example, the current incentives for the development of bioenergy may clash with the need to develop/produce new materials from biomass in a circular manner (OECD, 2018). Circular bioeconomy policies should imply that the co-products resulting from biomass processing should be kept within the (food) system as a priority (Ramirez, 2013; OECD, 2018), thus addressing the most pressing grand challenges, such as those associated with climate change, food, and energy security (Arsic et al., 2022). In the context of circular bioeconomy, trade-offs (e.g., conflict between industrial and environmental policy) should be well described and the use of co-products prioritized (OECD, 2018).

The basic concept of avoiding waste (“zero waste”) leads to the cascading of biomass, which involves the consequential production of raw materials and the generation of a suite of co-products used to manufacture subsequent products. Hence, priority and cascading concepts need to be considered to prioritize the use of biomass in a manner that limits conflicts and maximizes synergies (Kleinshmitt et al., 2017; OECD, 2018; Venugopal, 2022). Proper implementation of such an approach necessitates the development of an industrial symbiosis, through the organization of a network of local stakeholders, based on transversal and multidisciplinary activities (Reichel et al., 2016; Fraga-Corral et al., 2022).

## Food and Feed Safety

Sanitary risks are present throughout the entire production chain, including in the co-products themselves, during processing, storage, and distribution. Regulations and standards are in place to promote the safety of humans and animals, depending on the economic status of countries and consumer demand (Pinotti et al., 2021). Regulations may set strict limits for the presence of hazards, while some regulations may prohibit the use of co-products with the higher risks (Arujanan and Singaram, 2018; Pinotti et al., 2021; Van Raamsdonk et al., 2023).

The CODEX Alimentarius and ISO international standards are established for international food trade and serve as references for countries to formulate national regulations and standards. Standardization, labeling, certification, and notification systems are also imposed in various countries (Singh et al., 2021). In other countries, the lack of appropriate food safety assurance systems to protect public health is still a major challenge (Teferi, 2020). The harmonization of standards across national, regional, and industry sectors is also important to ensure that standards themselves do not become insurmountable barriers limiting circular bioeconomy approaches to valorize wastes and by-products (APEC, 2024).

Policy interventions and regulations are needed for the safe use of co-products in feed, with safety requirements being equivalent to conventional products, that they replace (Leiva et al., 2018; Sandström et al., 2022). Country-specific data are often needed to assess the health risk of new co-products (Javourez et al., 2021). To properly adress this challenge, a full coordination between technical experts and decision makers is necessary (Patel et al., 2023). It is recommended that producers of co-products for use in feed carry out the risk analysis and implement a Hazard Analysis and Critical Control Points approach covering both the manufacturing of the main product and all relevant co-products (FAO and IFIF, 2020).

Standards limiting the concentration of hazardous material in feed should include a risk assessment to determine if higher concentration of the co-product can be incorporated in the diet without increasing risk. These guidelines can be based on the “As Low As Reasonably Achievable” principles and require accurate analytical techniques to detect the hazard and its toxicological information in relation to the livestock species that it is fed to. It should be considered that the concentration of hazardous substances, such as toxins, may be higher in co-products compared to the main product. The proposed approach assesses overall exposure of the animal to the hazard and possible consequences of the co-product on animal, human, and environmental health.

Under the One Health concept, all types of hazard (for animals, humans, and environment) are assessed together, enabling an optimization of people, animal, and ecosystems (FAO, 2023) and has been proposed to be added to the three traditional pillars of economics, society, and environment (Perry et al., 2018).

Biological hazards relate to the presence of microbial pathogens in co-products that can cause diseases in livestock or be transferred to the consumer through animal-sourced food and cause zoonosis (e.g., *Salmonella* spp., *Campylobacter* spp., and *Escherichia coli*). They may enter the system either in the original product or post-processing, even if the main product is manufactured under hygienic conditions.

Mycotoxins are natural secondary metabolites produced by fungi, mainly *Aspergillus*, *Fusarium*, and *Penicillium.* Mycotoxins may be produced either during the growth of the crop in the field (e.g., deoxynivalenol, zearalenone, fumonisin) or during storage of the co-product (e.g., ochratoxin A, aflatoxin). The mycotoxin with the most significant impact on animal health and performance (mainly monogastric animals) are aflatoxin B1, ochratoxin A, deoxynivalenol, zearalenone, fumonisin, and some of these may also be a risk for the consumer of animal-sourced food, such as aflatoxin M1 (metabolite of aflatoxin B1 in milk) and ochratoxin A. To reduce the risk of mycotoxin contamination during storage, control strategies are important (Magan and Aldred, 2007; Neme and Mohammed, 2017).

Heavy metals may accumulate in the trophic chain and be toxic to livestock, humans, and the environment. They are usually poorly absorbed by the animals and the transfer of heavy metals to animal-sourced foods is relatively limited. However, some of them (e.g., cadmium) have a long half-life and may accumulate in some food (e.g., crustaceans). The concentration of heavy metals in co-products depends on the crop (growing area, fertilization) and of the manufacturing process of the main product and thus its co-product.

Dioxins and polychlorinated biphenyls are ubiquitous and may enter the food chain from various sources. They have the capacity to bioaccumulate in lipid-rich tissues of livestock when present in the feed. The primary source of exposure for consumers is related to the consumption of animal-sourced foods. It is therefore a priority hazard for feed and food safety (FAO and IFIF, 2020). Depending on the manufacturing process of the main products, the concentration of dioxins and polychlorinated biphenyls may increase, especially when oils and fats are extracted from the leading product and present in the co-product. Hence, the controls should focus on the manufacturing processes to prevent contamination from entering into the food chain. This was demonstrated when lime, contaminated by dioxins and polychlorinated biphenyls, was used in the drying process of citrus pulp (Malisch, 2017). Recycled oils and fats, hydrogenated fats, clay, guar gum, and treated wood shavings are examples of potential sources of contamination.

The potential impact of pesticides and veterinary drug residues associated with the use of co-products in feed is low. However, organochlorides may be problematic, as they may persist in the food chain. Furthermore, the use of co-products in feed presents some hazards for the environment, mainly due to their protein (nitrogen) and phosphorus content as well as the presence of zinc and copper (Hill et al., 2021). The nitrogen and phosphorus may lead, when in excess, to eutrophication, while zinc and copper may be toxic for the soils, when present at high concentration. It is therefore necessary to properly evaluate the nutritional composition of the co-products when using them in feed formulation to reduce the content of these minerals in manure.

## Planetary Boundaries and One Health

Circular bioeconomy approaches involve the safe reutilization of residual streams to close resource loops, providing many opportunities for transforming co-products into novel products for various industries. This increased level of movement of biomass across geographical areas and multiple industries poses some health and safety risks. In the case of livestock systems, manure streams are a pressing topic due to increasing policy and regulation limiting their management and reuse, due to concerns around environmental pollution (e.g., nitrogen leaching, the European Union Nitrates Directive) and greenhouse gas emissions. However, if properly managed through appropriate circular bioeconomy policy development and appropriate technologies, these resources could be valorized to produce a range of products such as bioenergy and biofertilizers.

Three key concepts can provide holistic and overlapping frameworks for an integrated, systems-based approach, to health and risk management for circular bioeconomy for livestock, humans, within the planetary boundaries, such as One Health, EcoHealth, and Planetary Health (Amanullah, 2024). While these concepts have different focus areas, collaboration across their relevant organizations is key to ensuring that their similar goals toward positive human, animal, and ecosystem health outcomes are met. The main differences have been summarized as "1) One Health involves collaborating disciplines working toward optimal health for the planet: its people, animals, and the environment", "2) EcoHealth is a systems-based approach to promoting health and well-being with a focus on social and ecological interactions"; and 3) Planetary Health aims to achieve health, well-being, and equity worldwide through attention to the human systems that shape the future of humanity and the Earth’s natural systems that define safe planetary boundaries (Hill-Cawthorne, 2019).

The planetary boundaries framework is a science-based analysis of the risk that human perturbations will destabilize ecosystems at the planetary scale (Steffen et al., 2015; Rockström et al., 2023). Nine planetary boundaries regulate the Earth’s stability and resilience within a safe operating space for humanity, including atmospheric aerosol loading, biogeochemical flows, biosphere integrity, climate change, freshwater use, land-system change, novel entities, ocean acidification, and ozone stratosphere depletion. The transgression of some of these planetary boundaries (e.g., climate change, biosphere integrity) impacts other planetary boundaries. Currently, six planetary boundaries have been transgressed beyond their safe operating space (Richardson et al., 2023). While circular bioeconomy approaches can be designed and applied to support the planetary boundaries, poorly managed approaches could cause maladaptive results (Table 1).

**Table 1.** Aligning circular bioeconomy approaches for livestock systems: example actions that may support or adversely affect planetary boundaries (Rockström et al., 2023)

Planetary boundary	Supportive circular bioeconomy approaches	Maladaptive circular bioeconomy approaches
Atmospheric aerosol loading	Applying bio-based fertilizers and soil amendments to improve soil structure and water holding capacity, reducing erosion and the likelihood of bushfires	Removing crop stubble, or residues to use for bioenergy or biomaterial synthesis, leaving soil exposed and prone to dust generation and erosion
Biogeochemical flows	Capturing nutrients from livestock-based organic residuals to return to agricultural lands to meet crop demand, reducing the need for external fertilizer P and N applications, thereby reducing net nutrient flows to waterways	Poor management of organic residues or biofertilizer applications may lead to excessive nutrient accumulation and discharge to the surrounding environments.
Biosphere integrity	Applying strategic grazing pressure to improve biodiversity outcomes in specific regions (e.g., reduce vegetation to manage weeds or reduce bushfire risk)	Geographic movement of livestock residuals and co-products without considering potential health risks may impact biodiversity (e.g., diseases)
Climate change	Developing upcycled feed ingredients to reduce GHG emissions through livestock diets	Poorly managed applications of biofertilizers or manure management can increase GHG and/or ammonia emissions.
Freshwater use	Prioritize capture of clean water, for reuse in upcycling livestock residuals and co-products (e.g., develop or apply technologies to separate nutrients and clean water from wastewaters)	Failing to consider freshwater requirements when designing circular bioeconomy manufacturing processes or products (e.g., separation technologies)
Land-system change	Upcycling co-products into novel feed ingredients may reduce the amount of land required for growing crops for use in feed.	Changes in land use disproportionately affect vulnerable groups (e.g., loss of income in developing economies).

The One Health Global Network employs an interconnected “whole of society” approach. However, its focus remains on veterinary and medical disciplines, with calls to incorporate aspects that are more a focus in EcoHealth and Planetary Health (e.g., climate change, biodiversity, agricultural systems, ecology, social science; Rabinowitz et al., 2018). The “One Health plan of action (2022 to 2026)” jointly created by the Food and Agriculture Organization, the United Nations Environmental Program, the World Health Organization, and the World Organization for Animal Health identified key activities, several of them having direct relevance to circular bioeconomy approaches:

-Controlling and eliminating zoonotic, neglected tropical, and vector-borne diseases,
-Strengthening the assessment, management, and communication of food safety risks,
-Curbing the silent pandemic of antimicrobial resistance, and
-Integrating the environment into One Health.

One Health action plans have been developed by more than 100 countries, on many topics including antimicrobial resistance. These action plans should be integrated within circular bioeconomy policies.

**Figure 1.**
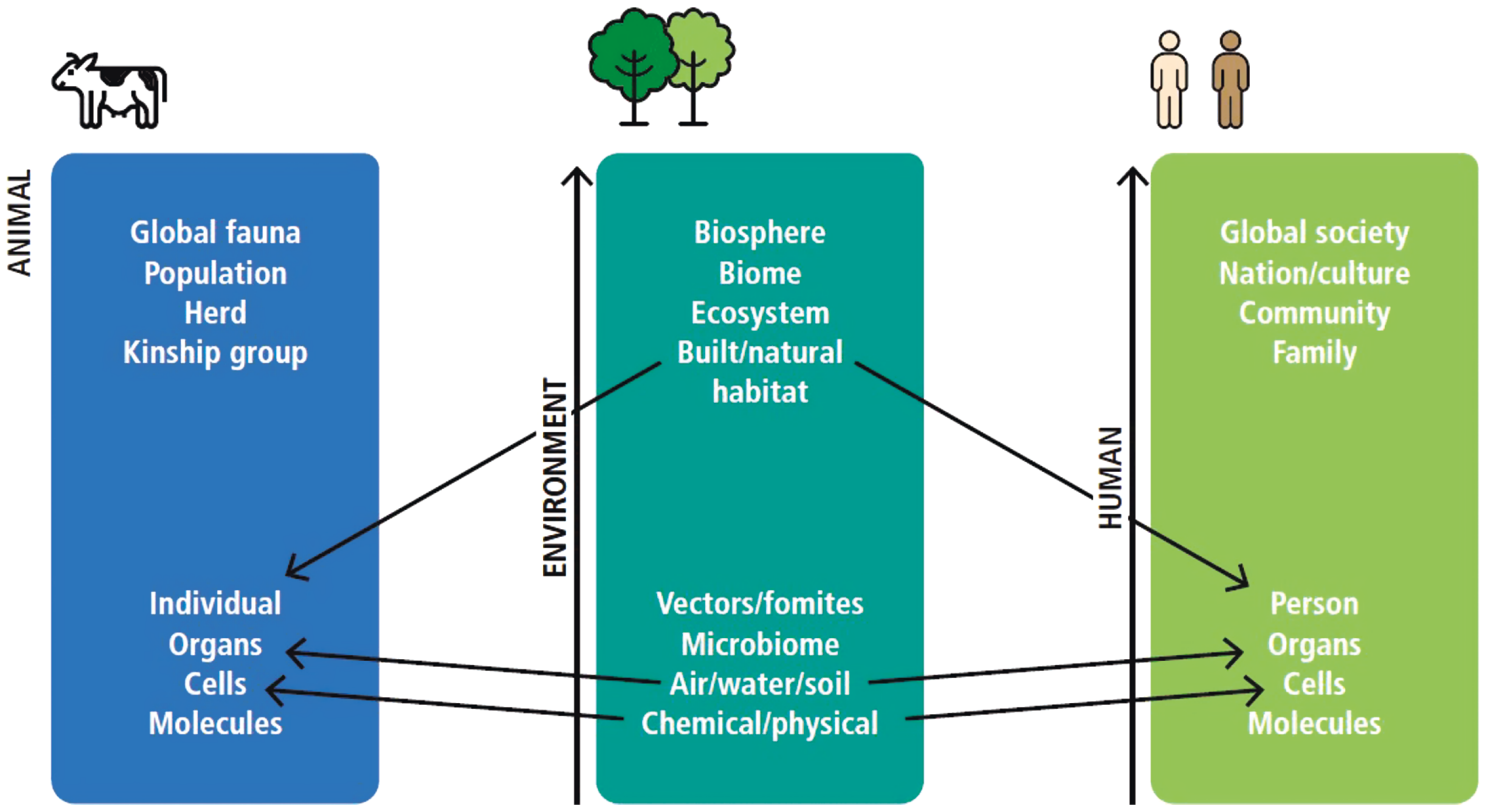
A One Health monitoring framework across animal, human, and environmental scales (Rabinowitz et al., 2018). Arrows indicate where monitoring efforts could link causal effects across and between scales within different systems.

A One Health system approach can also be used to develop monitoring frameworks for circular bioeconomy approaches. They may identify connections with and between scales of animal, human, and environmental systems, to identify health risks at macro scales (Figure 1).

Several frameworks can be used to develop supportive policies for a safe and sustainable circular economy, through a five-phase process that encompasses agenda setting, design, adoption, implementation, evaluation, and reform (Table 2).

**Table 2.** Five phases of the policy process according to Resnick et al. (2015, 2018) and roles for a potential role of FAO member states engagement

Policy process phase	Description of issues relevant to this policy process phase in the bioeconomy context	Potential roles for member states
Agenda setting	The concerns and priorities of individuals and communities for food and livelihood are frequently marginalized from decision-making processes. The importance of livestock production for exporting countries should not be forgotten.	Member states can help address imbalances in the prioritized issues in the political agenda or draw attention to the priorities of marginalized groups.
Design	The effort to apply the principles of the sustainable-livelihoods approach	Member States can assess the potential outcomes of alternative policy design options, highlight underlying biases, and inform multi-stakeholder deliberation.
Adoption	For member states that analyze ecological, economic, and social dynamics and must understand the institutional, legal, financial, and political context for decision-making, it may increase the possibilities of influencing the adoption of policies. These factors were critical, for example, in the alliances between civil society, nongovernmental organizations, and researchers that promoted the adoption of a human rights-based policy on the governance of the circular economy.	Member states can document and communicate the social distribution of anticipated costs and benefits of policy adoption as well as historical assistance to other groups to increase their voices and influence political decisions.
Implementation	Many policies fail due to inadequate capacity and commitment from government agencies at different levels, as well as inadequate capacity and commitment from community groups and civil society organizations. Policy implementation must consider not only the objective resources available to support implementation and the skills of the actors but also the alignment of values and interests that motivate collective action.	Associations in member states can assist with institutional capacity assessment required for implementing policies and support the development of capacities between societies.
Evaluation and reform	Evaluation research can indirectly influence the availability of resources, for example, by influencing a change in the political agenda that translates into budget decisions or market closures. A more direct opportunity, however, refers to evaluation research that influences political ideas and beliefs of advocates and veto players, particularly through well-substantiated evidence on progress and implications of policy implementation, challenges encountered, or new opportunities identified, and learned lessons. This includes studies evaluating national results, as well as comparative analysis between countries.	Participation in research can help assess the results of policy implementation, including the variety of results in different geographies and for other social groups, and generate lessons for adapting or reforming policy instruments.

## Conclusion

The current approach, consisting of developing separately bioeconomy and circular economy policies, is demonstrating a lack of coherence across relevant policy domains, inconsistencies, and potential contradictions, such as the incentives for biofuel production without considering other potential higher value products, or the lack of applying biomass cascading approaches to produce a suite of bioproducts. For an integrated approach, the development of circular bioeconomy policies, including livestock production systems, should be considered in the future. These bioeconomy policies would be developed within the context of the One Health, EcoHealth, and Planetary Health approaches, to ensure safe, sustainable, and efficient use of biological resources present in by-products and residues that are currently largely managed as waste.

To ensure the safety of the food chain, the co-products used in feed should be monitored and controlled as conventional feed ingredients. Hence, current regulatory limits and standards on contaminants should be applied to co-products, considering their specificities and in relation with their manufacturing processes. Furthermore, industries, responsible for producing the main product (and by- and co-products), should include an assessment of their co- and by-products in the Hazard Analysis and Critical Control Points systems, to ensure proper control and monitoring of potential contaminants for use in other valuable bioproduct streams.

## About the Authors

**Maja Arsic** is a Research Scientist at CSIRO Agriculture & Food. She holds a PhD in Plant Nutrition (University of Copenhagen/University of South Australia) with a background in environmental chemistry (Griffith University). She works across the science–policy interface to enable circular bioeconomy transition pathways for sustainable agrifood systems, and is a national Science & Technology Australia STEM Ambassador. Maja currently works with a range of stakeholders and sectors, including aquaculture, water management, bioenergy, and livestock industries, to design and deliver pathways toward achieving circularity at local, regional, and international scales.

**Eleazar Gonzalez** is an Assistant Professor and State Extension Specialist in Agricultural Economics and Marketing in Small Sustainable Farms at Lincoln University of Missouri. Over the last 15 years, he has been working to build sustainable agricultural capacity among small farmers and ranchers, with a focus on developing an understanding of sustainable agribusiness models, implementing sustainable practices, and assessing the economic, social, and environmental impacts. His multidisciplinary background, encompassing economics, sociology, and agronomy, has enabled him to collaborate with private and public organizations to enhance the sustainability of small-scale farmers and ranchers.

**Jesus Mendez** is a retired professional after 35 years working in animal nutrition and livestock production at a leading cooperative in Spain. He holds a PhD in animal nutrition from the Polytechnic University of Madrid (Spain). He has collaborated on multiple research projects, publishing several papers, and participated in the development of some prestigious animal feeding tables. Jesus currently focuses on sustainability issues, collaborating with international organizations (FAO, UNECE, FEFAC EC).

**Duncan Rowland** is an experienced CEO who has a breadth of experience in management, governance, and strategic planning across a range of sectors (commercial, government, research, adoption, and not-for-profit). As such, he has been responsible for leading organizations and programs, including managing financial, human, and asset resources for a range of projects, enterprises, and businesses. I have found that it is essential to underpin these business activities with good planning, policy development, technical knowledge, and robust review methodologies. Continual improvement is essential to building the business and refining the business model.

**Philippe Becquet** is a consultant for regulatory and sustainability topics. He provides advice to the feed industry. Agronomist and animal nutritionist as a background, Philippe Becquet has worked for more than 40 years in the feed industry at various positions and in various companies, accumulating experience and expertise in livestock production systems and feed industry. He has also been active in feed industry associations such as IFIF (International Feed Industry Federation) and has contributed as an expert in the development of the Livestock Environmental Assessment and Performance (LEAP) guidelines on feed additives, the report on methane, and more recently, the report on the role of livestock in circular bioeconomy.

**Corresponding author**: becquet@aphaia.fr
